# Trait-dependent associations between early- and late-life reproduction in a wild mammal

**DOI:** 10.1098/rsbl.2023.0050

**Published:** 2023-07-12

**Authors:** Chris McKenna-Ell, Sanjana Ravindran, Jill G. Pilkington, Josephine M. Pemberton, Daniel H. Nussey, Hannah Froy

**Affiliations:** Institute of Ecology and Evolution, School of Biological Sciences, University of Edinburgh, Edinburgh EH9 3FL, UK

**Keywords:** senescence, ageing, life-history trade-off, Soay sheep *Ovis aries*

## Abstract

Early- versus late-life trade-offs are a central prediction of life-history theory that are expected to shape the evolution of ageing. While ageing is widely observed in wild vertebrates, evidence that early–late trade-offs influence ageing rates remains limited. Vertebrate reproduction is a complex, multi-stage process, yet few studies have examined how different aspects of early-life reproductive allocation shape late-life performance and ageing. Here, we use longitudinal data from a 36-year study of wild Soay sheep to show that early-life reproduction predicts late-life reproductive performance in a trait-dependent manner. Females that started breeding earlier showed more rapid declines in annual breeding probability with age, consistent with a trade-off. However, age-related declines in offspring first-year survival and birth weight were not associated with early-life reproduction. Selective disappearance was evident in all three late-life reproductive measures, with longer-lived females having higher average performance. Our results provide mixed support for early–late reproductive trade-offs and show that the way early-life reproduction shapes late-life performance and ageing can differ among reproductive traits.

## 1. Introduction

Senescence, the age-related decline in physiological function and reproductive value, is prevalent across the tree of life [1]. Despite its ubiquity, senescence is a hugely diverse process that varies across many biological levels: among cells, tissues and organs, through different phenotypic traits, to variation between individuals, sexes, populations and species [2–5]. Trade-offs between early-life reproduction and the maintenance of physiological and reproductive function in later adulthood form a cornerstone of the antagonistic pleiotropy and disposable soma theories on the evolution of ageing [6–8]. This theory predicts that investment of limited resources in energetically demanding activities such as growth and reproduction may come at a cost in terms of somatic maintenance [9,10]. Variation in allocation to reproduction in early life is therefore expected to be a key driver of among-individual variation in the onset and rate of senescence [6,11].

In vertebrates, reproduction is a composite trait which can be conceptualized sequentially, from the probability of breeding, successful mating, and fertilization to offspring production, provisioning and successful recruitment to the breeding population [12]. While there is widespread evidence that reproductive traits show senescent declines in wild vertebrates [13,14], there is also increasing evidence of asynchrony of senescence among reproductive traits within populations [3,15,16]. While early–late trade-offs are well documented in laboratory model organisms [17], evidence from natural systems remains mixed [18–21] as heterogeneity among individuals in resource acquisition and selective disappearance can mask both trade-offs and ageing patterns [22–24]. Recent studies confirm that where longitudinal lifelong data are available and appropriate statistical controls are used to account for heterogeneity, early–late trade-offs can be detected in wild populations [11,25,26]. Yet, few studies have tested whether and how trade-offs between early- and late-life reproduction might differ across reproductive traits.

In this study, we used 36-years of longitudinal data from an unmanaged population of Soay sheep on St Kilda, Scotland, to test whether reproduction in early life was associated with increased rates of senescence in three components of female reproduction: breeding probability, offspring birth weight and offspring first-winter survival, thereby testing a core prediction of some of the key evolutionary theories of ageing in the wild.

## 2. Methods

### (a) Study system

Longitudinal monitoring of Soay sheep (*Ovis aries*) on the island of Hirta in the St Kilda archipelago, Scotland, has been ongoing since 1985. Ewes conceive during the annual autumn rut and gestate over the winter months, giving birth to singleton or twin lambs in late March–April. Ninety-five per cent of lambs born in the study are caught, sampled and uniquely tagged shortly after birth [27]. Parentage is determined through a combination of field observation and genetic methods [27]. Females sexually mature during their first year, and 44% of those that survive to 1 March in the year following birth give birth to a live lamb around their first birthday. The vast majority of mortality occurs in late winter and early spring (89% deaths occur January–April). Mortality searches during this period in combination with 30 censuses conducted throughout the year mean that the fate of individuals is known with a high degree of accuracy. Senescent declines have been documented in morphological, physiological and demographic traits including components of female reproductive performance [3,28,29]. In this study, we used data on known aged females collected between 1985 and 2020.

### (b) Reproduction in early and later life

We considered three key components of annual reproductive performance in later life:

— *Breeding probability (0/1)*: probability of giving birth to a live lamb conditional on a female being alive on 1 March year *t* (*n* = 3173 observations of 762 females)
— *Offspring first-winter survival (0/1)*: probability of a female having at least one surviving yearling on 1 May year *t* + 1 conditional on giving birth to a live lamb in year *t* (*n* = 2573 of 714)
— *Offspring birth weight*: weight of lamb on first capture to the nearest 0.1 kg, corrected for age at capture (in days) (*n* = 2317 of 649).

We did not include twinning probability, the other major component of female reproductive performance, because the rate of twinning is low (approx. 11%), and it does not show signs of senescence (electronic supplementary material, figure S1). Based on the population-level ageing trajectories of our chosen traits (electronic supplementary material, figure S1), we designated ages 5 years and older as ‘later life’. This captured the peak performance for all three traits while minimizing nonlinearity in the subsequent decline. We re-ran our analyses using cut-offs at age 4 and 6 to check that our results were not dependent on the precise threshold used.

We used two traits to capture variation in early-life reproduction:

— *Bred as a yearling (0/1)*: whether a female born in year *t* gave birth to a live lamb in year *t* + 1. Note that females in this age class never have twins.
— *Early-life recruitment*: the number of offspring a female successfully raised through their first winter (to 1 May the year following birth) in the first 4 years of her life (i.e. lambs born when she was aged 1–3 years).

The distribution of early-life traits for individuals included in the analyses of later life reproduction are shown in the electronic supplementary material, figure S2. There was little overlap between the traits, with females that bred as yearlings having a similar distribution of early-life recruitment compared to those that did not (electronic supplementary material, figure S2).

### (c) Statistical analysis

We modelled reproductive performance in later life as a function of early-life reproduction using (generalized) linear mixed-effects models. Breeding probability and offspring first-winter survival were modelled as binary traits with a binomial error distribution and logit link function. Offspring birth weight was modelled as a Gaussian trait with identity link function. We included a linear effect of age in all models to capture senescence in reproductive performance beyond the age of 5. Age at last observation was included as a fixed covariate to account for the selective disappearance of shorter-lived individuals that may differ in their performance throughout later life [23]. We used age at last observation rather than longevity so we could include individuals that were still alive in our analyses (less than 10% of observations). The inclusion of this term meant that we could interpret the effect of age as a within-individual effect in our models. To test for associations between early-life reproduction and average performance in later life, we included whether a female bred as a yearling as a two-level fixed factor and early-life recruitment as a fixed covariate. We also included each of these terms in a two-way interaction with age to test whether the rate of ageing was dependent on reproduction in early life. In our models of offspring birth weight, we included offspring age at time of capture in days as a fixed covariate to account for the rapid growth that occurs during this period, offspring sex and whether they were a twin as two-level fixed factors as these factors are known to influence birth weight. All models included female identity and observation year as random intercept terms to account for repeated measures of individuals and years. We also included female cohort as a random intercept term to account for the fact that females born in the same year experienced similar conditions during early life. All terms were retained in the model regardless of statistical significance. Models were run using *lme4* [30] in the R programming environment (v. 4.2.2; [31]).

## 3. Results

Breeding probability, offspring first-winter survival and offspring birth weight all declined with increasing age in females aged 5 and older (table 1; electronic supplementary material, figure S3). Selective disappearance was evident in all three reproductive traits with females that were older at last observation having higher average trait values (table 1; electronic supplementary material, figure S3).

**Table 1.** Fixed and random effect estimates from (generalized) linear mixed-effects models of (A) breeding probability; (B) offspring first-winter survival and (C) offspring birth weight in female Soay sheep aged 5 years and older. Breeding probability and offspring survival were modelled using a binomial error distribution and logit link function; offspring birth weight was Gaussian with an identity link function. Estimates are given on the latent scale. Fixed effects where *p* < 0.05 are highlighted in italics.

random effects	variance	fixed effects	estimate	s.e.	*p*-value
(A) breeding probability					
female identity	3.061	intercept	3.287	0.549	<0.001***
year	0.514	*age*	*−0.487*	*0.060*	<0.001***
female cohort	0.147	*age at last observation*	*0.188*	*0.054*	<0.001***
		*bred as yearling*	*2.077*	*0.587*	<0.001***
		*early-life recruitment*	*0.948*	*0.464*	0.041*
		*age : bred as yearling*	*−0.210*	*0.075*	0.005**
*n* *=* *3173 observations of 762 females*		age : early-life recruitment	0.002	0.058	0.972
(B) offspring first-winter survival					
female identity	0.207	intercept	0.077	0.442	0.862
offspring birth year	1.491	*age*	*−0.232*	*0.054*	<0.001***
female cohort	0.004	*age at last observation*	*0.089*	*0.032*	0.006**
		bred as yearling	−0.468	0.415	0.260
		*early-life recruitment*	*0.869*	*0.309*	0.005**
		age : bred as yearling	0.038	0.060	0.527
*n* *=* *2573 observations of 714 females*		age : early-life recruitment	−0.063	0.043	0.149
(C) offspring birth weight					
female identity	0.066	intercept	3.013	0.097	<0.001***
offspring birth year	0.057	*offspring capture age*	*0.112*	*0.003*	<0.001***
female cohort	0.004	*offspring sex*	*0.106*	*0.016*	<0.001***
residual	0.115	*offspring twin status*	*−0.826*	*0.020*	<0.001***
		*age*	*−0.061*	*0.009*	<0.001***
		*age at last observation*	*0.016*	*0.007*	0.031*
		bred as yearling	0.053	0.070	0.443
		early-life recruitment	0.019	0.050	0.705
		age : bred as yearling	0.005	0.010	0.572
*n* *=* *2317 observations of 649 females*		age : early-life recruitment	0.005	0.007	0.432

**Figure 1.**
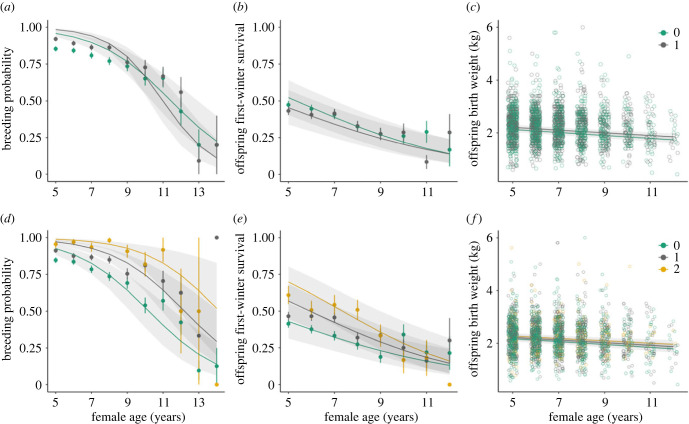
Associations between early-life reproduction (breeding as a yearling and early-life recruitment) and age-specific reproduction in later life in Soay sheep. Predictions and 95% confidence intervals from (generalized) linear mixed-effects models of (*a,d*) female breeding probability; (*b,e*) offspring first-winter survival and (*c,f*) offspring birth weight in female sheep aged 5 years and older. Plots show the effect of female or maternal age along with the additive or interactive effects of early-life reproduction. Plots (*a–c*) show the effect of breeding as a yearling (grey) versus not (green); plots (*d–f*) show the effect of successfully recruiting 0, 1 or 2 offspring in the first 4 years of life (0 = green; 1 = grey; 2 = yellow). Lines show the model predictions with 95% confidence intervals in shaded grey; points show the raw data means and standard errors (plots (*a*), (*b*), (*d*) and (*e*)) or raw data (plots (*c*) and (*f*)). Full models are shown in table 1.

Reproduction in early life was positively associated with later life breeding probability: females who bred as yearlings and those who successfully recruited more offspring in their first 4 years of life were more likely to breed annually from 5 years of age (table 1A; electronic supplementary material, figure S3A). The rate of senescence in breeding probability was associated with early-life reproduction: females who bred as yearlings suffered more rapid declines in breeding probability than those that did not (interaction between female age and bred as yearling = −0.210 ± 0.075, *p* = 0.005; table 1A; figure 1*a*). By contrast, successfully raising more offspring to recruitment was not associated with more rapid ageing in breeding probability (table 1A; figure 1*d*).

Females with higher early-life recruitment success also had higher offspring survival in later life, but there was no association between breeding as a yearling and late-life offspring survival (table 1B; electronic supplementary material, figure S3B). There was no association between reproduction in early life and offspring birth weight in later life (table 1C; electronic supplementary material, figure S3C). The rate of ageing in offspring first-winter survival and offspring birth weight was not dependent on whether a female bred as a yearling or their recruitment success during early life (table 1B,1C: figure 1*b*,*c*,*ef*). Our results were not dependent on the age threshold used to define later life: excluding observations from females below 4 or 6 years of age did not qualitatively change our results (electronic supplementary material, table S1).

## 4. Discussion

We found mixed evidence that early–late trade-offs drive variation in the rate of reproductive senescence in female Soay sheep. Females who bred as yearlings had a higher probability of breeding at the age of 5 years, but this was associated with a faster rate of age-related decline in breeding probability (as indicated by the crossed lines in figure 1*a*). This suggests a trade-off in performance between early and later life and supports key theoretical predictions that among-individual variation in the rate of ageing may be driven by differences in early-life reproductive allocation. However, breeding as a yearling was not associated with the rate of senescence in offspring first-winter survival or offspring birth weight (figure 1*b,c*). Similarly, the number of offspring that a female managed to successfully recruit during early life was not associated with the rate of senescence in any of our reproductive traits (figure 1*d,e,f*). Overall, our results therefore suggest that reproduction in early life is not a key driver of among-individual variation in reproductive senescence in this system.

In line with previous findings, there was evidence for senescence in multiple components of female reproduction (table 1) [3,28]. These declines were substantial: the probability of giving birth to a live lamb declined from a mean of 0.96 (95%CI 0.93–0.97) aged 5 to 0.32 (0.18–0.50) aged 13 years; the probability of such a lamb surviving its first winter declined from 0.52 (0.40–0.64) to 0.11 (0.06–0.20), and mean singleton lamb weight declined from 2.21 kg (2.11–2.31) to 1.80 kg (1.66–1.93). Senescent declines were therefore apparent at multiple stages of reproduction, from successful oocyte production and gestation through to lactation and offspring provisioning. Life-history theory of ageing predicts that these declines may reflect a delayed cost of investment in reproduction, with females that breed successfully in the first few years of life allocating relatively less to somatic maintenance and consequently suffering more rapid senescence [9]. This pattern has been observed in some wild vertebrate study systems [20,25,26], but not in others [18,19,21]. Our results suggest that the detectability of long-term costs of early-life reproductive allocation may depend on the reproductive trait being investigated, which may help explain the mixed evidence from field studies to date. Furthermore, there is mounting evidence for asynchrony in the patterns of senescence across reproductive traits in wild vertebrates [3,32,33,34]. Variation in the manifestation of early versus late trade-offs among reproductive traits may help explain some of this asynchrony.

Overall, our metrics of early-life reproduction tended to be positively associated with average reproductive performance in later life (table 1). This suggests heterogeneity among females that persists over the lifetime, potentially masking trade-offs between early and later life [22,35]. Such ‘carry over’ or ‘quality’ effects are widely observed in wild systems and may be driven by genetics, persistent early-life effects, or aspects of an individual's environment that remain constant throughout life such as home range quality [17,36]. Such heterogeneity was also evident as selective disappearance in all three traits in later life (table 1), further highlighting the importance of longitudinal data and statistically controlling for such effects in the study of reproductive ageing [23,24]. Short-term reproduction–survival trade-offs may also play an important role in our failure to observe predicted early versus late reproductive trade-offs, if individuals that invest heavily in early-life reproduction are less likely to survive to senescent ages. Indeed, reproduction in yearlings is strongly environment-dependent and associated with reduced yearling survival in this population [37]. Our analyses included only females that survived to age 5 and so would not capture short-term survival costs of early reproduction. Overall, our results do not support an important role for early–late reproductive trade-offs in driving variation in reproductive ageing in wild Soay sheep. However, they do highlight the need for increased consideration of different reproductive traits in studies of reproductive ageing and early–late trade-offs, and the importance of understanding the drivers of among-individual heterogeneity in adult reproductive performance in wild populations.

## Data Availability

Data and accompanying description are available from the Dryad Digital Repository: https://doi.org/10.5061/dryad.stqjq2c7s [38]. The data are provided in the electronic supplementary material [39].
